
JOURNAL INFORMATION
==============================
NLM Title Abbreviation: ScientificWorldJournal
Journal ID: ScientificWorldJournal
NLM Journal ID: 101131163
Journal ID: TSWJ

The Scientific World Journal

ISSN: 2356-6140
EISSN: 1537-744X
Publisher: Wiley

ARTICLE INFORMATION
==============================
PMCID: PMC6084100
DOI: 10.1100/tsw.2001.422
Article ID: 463472
Article version: 1
Subjects: Extended Abstract

Pharmacological Differences between Human and Rat CGRP Receptors Are Determined by RAMP1

Kane S. 1
Mallee J. 1
Salvatore C. 1
LeBourdelles B. 2
Koblan K. 1
1 Merck Research Laboratories, Department of Pharmacology, West Point, PA 19486, USA
2 Merck Research Laboratories, Neuroscience Research Center, Harlow, Essex CM20, UK
Publication date: 2001
Electronic publication date: 2001 Dec 8
Volume: 1
First page: 28
Last page: 28
Copyright: Copyright © 2001 S. Kane et al.
Copyright year: 2001
License: This is an open access article distributed under the Creative Commons Attribution License, which permits unrestricted use, distribution, and reproduction in any medium, provided the original work is properly cited.
License URL: https://creativecommons.org/licenses/by/3.0/

==============================
4th International Meeting on Calcitonin Gene-Related Peptide (CGRP)
*TheScientificWorld* (2001) 1(S1), 28
ISSN 1532-2246; DOI 10.1100/tsw.2001.422

### PHARMACOLOGICAL DIFFERENCES BETWEEN HUMAN AND RAT CGRP RECEPTORS ARE DETERMINED BY RAMP1

S. Kane[1], J. Mallee[1], C. Salvatore[1], B. LeBourdelles[2], and K. Koblan[1]
[1]Merck Research Laboratories, Department of Pharmacology, West Point, PA 19486; [2]Merck Research Laboratories, Neuroscience Research Center, Harlow, Essex CM20 U.K.

### INTRODUCTION

Several small molecule CGRP receptor antagonists have now been reported and their effects profiled in a variety of *in vivo* assays. Of particular interest is the observation that some antagonists display marked species selectivity. Doods and coworkers[1] reported that BIBN4096BS exhibited 200-fold higher affinity for human and marmoset CGRP receptors, than for receptors from rat, dog, guinea pig and rabbit. In addition, Edvinsson and colleagues[2] reported that compound 1 was a significantly more potent antagonist on human cerebral arteries than on guinea pig arteries. In the present study, we sought to identify the molecular basis for this profound species selectivity.

### METHODS

Hybrid CGRP receptors were constructed by cotransfection of either human or rat CRLR with human or rat RAMP1 in 293 EBNA cells. Chimeric RAMP1 constructs were prepared by replacing the first 66 amino acids (chimera 1) or first 112 amino acids (chimera 2) of rat RAMP1 with the corresponding human sequence. Human or rat CRLR was cotransfected with either the native or chimeric RAMP1. Membranes were prepared and employed for competition binding and deglycosylation experiments.

### RESULTS

The $IC_{50}$s of BIBN4096BS and compound 1 for displacement of $^{125}$I-hCGRP from hCRLR/rRAMP1 membranes were similar to that for rCRLR/rRAMP1. The affinity of $CGRP_{8-37}$ was unaffected by the species origin of RAMP1. The $IC_{50}$s of BIBN4096BS and compound 1 for rCRLR/chimera1 were similar to that for rCRLR/rRAMP1, whereas rCRLR/chimera 2 yielded $IC_{50}$s similar to that of rCRLR/hRAMP1.

Coexpression of rCRLR with either human or rat RAMP1 resulted in the production of a mature glycoprotein.

### CONCLUSION

The high affinity of BIBN4096BS and compound 1 for hCRLR/hRAMP1 was dictated strictly by hRAMP1. The region between amino acids 66-112 was critical for determining the pharmacology of these small molecule antagonists. The exact molecular mechanisms by which RAMP1 modulates antagonist binding sites remain to be elucidated.

### REFERENCES

1. Doods, H., Hallermayer, G., Wu, D., Entzeroth, M., Rudolf, K., Engel, W., and

Eberlein, W. (2000) Pharmacological profile of BIBN4096BS, the first selective small molecule CGRP antagonist. Br. J. Pharmacol. 129, 420–423.
2. Edvinsson, L., Sams, A., Jansen-Olesen, I., Tajti, J., Kane, S.A., Rutledge, R.Z., Koblan, K.S., Hill, R.G., and Longmore, J. (2001) Characterisation of the effects of a non-peptide CGRP receptor anagonist in SK-N-MC cells and isolated human cerebral arteries. Eur. J. Pharmacol. 415, 39–44.
